# MammaPrint predicts chemotherapy benefit in HR+HER2- early breast cancer: FLEX Registry real-world data

**DOI:** 10.1093/jncics/pkaf079

**Published:** 2025-08-11

**Authors:** Adam M Brufsky, Kent F Hoskins, Henry J Conter, Pond Kelemen, Mehran Habibi, Laila Samian, Rakshanda L Rahman, Laura Lee, Eduardo C Dias, Regina Hampton, Beth A Sieling, Cynthia R Osborne, Eric Brown, Jailan A Elayoubi, Priyanka Sharma, Jayanthi Ramadurai, Laurie Matt-Amaral, Alfredo A Santillan, Sasha Davis, Philip Albaneze, Harshini Ramaswamy, Nicole Chmielewski-Stivers, Andrea Menicucci, William Audeh, Pat Whitworth, Nathalie Johnson, Joyce O’Shaughnessy

**Affiliations:** Department of Medicine, University of Pittsburgh Medical Center, Pittsburgh, PA, United States; College of Medicine, University of Illinois Cancer Center, Chicago, IL, United States; Department of Oncology, William Osler Cancer Centre, Brampton, ON, Canada; Breast Surgical Oncology, Northwell Health, Sleepy Hollow, NY, United States; Breast Surgical Oncology, Northwell Health, Staten Island, NY, United States; Surgical Oncology, Baptist Health MD Anderson Cancer Center, Jacksonville, FL, United States; Department of Surgery, Texas Tech University Health Sciences Center School of Medicine, Lubbock, TX, United States; Surgical Oncology, Comprehensive Cancer Center, Palm Springs, CA, United States; Medical Oncology, Tennessee Oncology, Nashville, TN, United States; Surgical Oncology, Luminis Health, Lanham, MD, United States; Surgical Oncology, Trinity Health of New England, Southbury, CT, United States; Medical Oncology, Texas Oncology, Dallas, TX, United States; Surgical Oncology, Comprehensive Breast Care, Troy, MI, United States; Medical Oncology, Karmanos Cancer Institute, Detroit, MI, United States; Medical Oncology, University of Kansas Medical Center, Kansas City, KS, United States; Medical Oncology, Affiliated Oncologists, LLC, Chicago Ridge, IL, United States; Medical Oncology, Cleveland Clinic, Akron, OH, United States; Surgical Oncology, Texas Oncology, San Antonio, TX, United States; Medical Oncology, Texas Oncology, Tyler, TX, United States; Breast Surgical Oncology, Roper St Francis Healthcare, Charleston, SC, United States; Medical Affairs, Agendia, Inc, Irvine, CA, United States; Medical Affairs, Agendia, Inc, Irvine, CA, United States; Medical Affairs, Agendia, Inc, Irvine, CA, United States; Medical Affairs, Agendia, Inc, Irvine, CA, United States; Surgical Oncology, Nashville Breast Center, Nashville, TN, United States; Surgical Oncology, Legacy Health System, Portland, OR, United States; Medical Oncology, Baylor University Medical Center, Texas Oncology, Sarah Cannon Research Institute, Dallas, TX, United States

## Abstract

**Background:**

Gene expression assays help personalize adjuvant chemotherapy decisions for hormone receptor-positive, HER2-negative (HR+HER2-) early breast cancer (EBC). The 70-gene risk of distant-recurrence signature, MammaPrint, demonstrated clinical utility in guiding chemotherapy de-escalation in genomically low risk patients in the MINDACT trial. This study evaluates MammaPrint as a continuous predictor of chemotherapy benefit in HR+HER2- EBC using real-world data (RWD) from the FLEX Registry.

**Methods:**

The study evaluated 1002 patients treated with endocrine therapy (ET) only or ET with chemotherapy (ET+CT) enrolled in FLEX (NCT03053193) with 5-year median follow-up. Propensity-score matching balanced treatment groups by menopausal status, T-stage, and nodal status. The primary endpoint was distant recurrence-free interval (DRFI). Regression and Cox proportional hazards models assessed chemotherapy benefit across MammaPrint Index (MPI) risk.

**Results:**

Most patients were postmenopausal (70.1%), node-negative (70.0%), and had grade 2 tumors (51.2%). The regression models showed that MPI strongly predicted 5-year DRFI in ET only (R^2^ = 0.99, *P < *.001) and ET + CT (R^2^ = 0.90, *P < *.001) groups, corresponding to an average absolute chemotherapy benefit of 5.6% in High 1 and 10.9% in High 2. Minimal improvement in DRFI with chemotherapy was observed for Low (1.7%) and UltraLow (<1.0%) risk groups. A multivariate Cox model with an MPI-by-treatment interaction term demonstrated that increasing MPI risk was associated with greater chemotherapy benefit on DRFI (HR = 0.15, *P = *.047). Chemotherapy benefit was significantly associated with premenopausal status, but not age, T-stage, nodal status, or grade.

**Conclusions:**

These RWD from the FLEX Registry demonstrate that MPI is predictive of both DRFI prognosis and chemotherapy benefit in HR+HER2- EBC. (NCT03053193)

## Introduction

The hormone receptor-positive, human epidermal growth factor receptor 2-negative (HR+HER2-) subtype accounts for approximately 70% of all female breast cancers (BCs).^1^ Due to the high heterogeneity of HR+HER2- BC, the use of adjuvant chemotherapy is based on a multitude of patient factors.^2^^,^^3^ Gene expression profiling assays have become standard-of-care diagnostic tools to aid in adjuvant chemotherapy planning decisions. The 70-gene assay, MammaPrint, is a genomic signature that assigns early-stage BCs as having a MammaPrint Index score of UltraLow, Low, High 1 (H1), or High 2 (H2) risk of distant recurrence.

The phase III, randomized MINDACT (NCT00433589) trial found that early-stage BC patients with clinical high risk and MammaPrint Low or UltraLow Risk BC had excellent 8-year distant recurrence-free survival (DRFS) when treated with endocrine therapy alone, without chemotherapy.^4^^,^^5^ However, MINDACT was not designed or statistically powered to identify which patients benefited from chemotherapy based on MammaPrint risk; exploratory analyses of this question were limited by low sample size and event rates. Therefore, studies investigating whether MammaPrint can predict neoadjuvant/adjuvant chemotherapy benefit are needed.

Real-world evidence (RWE) derived from real-world data (RWD) can address critical questions in oncology that cannot be practically addressed through randomized controlled clinical trials (RCTs).^6-8^ The FLEX Registry trial (NCT03053193) was initiated in 2017, with the goal of enrolling 30 000 early-stage BC patients followed for 10 years to correlate detailed clinical RWD with whole-transcriptome tumor profiling, including standard-of-care MammaPrint testing.^9^

The primary objective of this FLEX trial analysis was to estimate adjuvant chemotherapy benefit as a function of the continuous MammaPrint Index using 5-year real-world outcome data from a propensity score-matched cohort of patients with HR+HER2- early-stage BC treated with adjuvant endocrine therapy (ET) only or with both ET and chemotherapy (ET+CT).

## Material and methods

### Study design and patient population

FLEX (MammaPrint, BluePrint, and Full-Genome Data Linked with Clinical Data to Evaluate New Gene EXpression Profiles: An Adaptable Registry) is an ongoing, prospective, observational, real-world evidence registry trial. The study is enrolling patients aged ≥18 years with stage I-III invasive BC whose primary cancers undergo standard-of-care MammaPrint genomic testing, with or without BluePrint molecular subtyping, in addition to whole transcriptome sequencing. The protocol was approved by Institutional Review Boards at all participating institutions and is registered with ClinicalTrials.gov (NCT03053193). FLEX follows ethical principles outlined in the Declaration of Helsinki,^10^ adheres to international Good Clinical Practice (GCP) guidelines,^11^ and is approved by the Institutional Review Board of WCG (protocol code #2017003). All participants provided informed consent for study participation and full genome and clinical data collection. Systemic therapy decisions were made at the physicians’ discretion.

The current analysis included FLEX trial participants diagnosed within 93 US institutions between 2012 and 2021 with HR+HER2- BC with a median follow-up of 5 years postdiagnosis (*n* = 1407; data cutoff: April 2, 2024). Within this nonrandomized population, 859 patients received ET only, and 548 patients received ET+CT. To ensure that the 2 cohorts were comparable regarding clinical characteristics (Table S1), nearest neighbor propensity-score matching was performed to balance menopausal status, tumor stage, and lymph node status. Matching was conducted using the matchit() function in R, employing a caliper of 0.5 yielding 501 patients per group (*n* = 1002 total).

### Pathological and genomic evaluation

MammaPrint is a microarray-based 70-gene expression assay performed on RNA extracted from formalin-fixed, paraffin-embedded (FFPE) tissue with a minimum 30% tumor cell content sent to Agendia, Inc., according to established protocols as previously described.^12-14^ Briefly, RNA was converted to cDNA, amplified, labeled, and hybridized onto Agendia’s custom-designed diagnostic arrays (Agilent Technologies). Full-genome and MammaPrint Index results were generated independent of clinical and pathological data and classified tumors as having a Low Risk (MammaPrint Index >0) or High Risk (MammaPrint Index ≤0) of distant recurrence. The MammaPrint Index was further stratified into UltraLow (1.000 to 0.356), Low (0.355 to 0.001), H1 (0 to -0.570), and H2 (-0.571 to -1.000) Risk. Hormone receptor and HER2 status was locally assessed by immunohistochemistry (IHC) and/or fluorescence in situ hybridization (FISH) according to research sites’ standard guidelines.

### Statistical analysis

Descriptive statistics were used to summarize age, menopausal status, race, tumor stage (T), lymph node (LN) status, tumor grade (G), and MammaPrint results. Differences in clinical characteristics between the nonrandomized, propensity score-matched treatment groups were assessed using χ^2^ or Fisher exact tests for categorical variables and *t*-tests for continuous variables. A sensitivity analysis evaluated the distribution of menopausal status, tumor grade, nodal status, and tumor stage across the MammaPrint Index continuum by χ^2^ test. A Cramer’s V test assessed collinearity between clinical tumor grade and MammaPrint Risk categories.

The primary endpoint was distant recurrence-free interval (DRFI), defined as the time from diagnosis to distant recurrence or BC-specific death, per STEEP 2.0 criteria.^15^ To evaluate 5-year DRFI as a continuous function across the MammaPrint Index, the Index range (-1 to +1) was segmented to optimize empirical risk estimation for each treatment group (ET only and ET+CT). Empirical 5-year DRFI was calculated for each segment using Kaplan-Meier analysis and a quadratic polynomial regression was applied to model the relationship between the MammaPrint Index and DRFI, generating risk curves for each treatment group. Bootstrapping was used to precisely estimate confidence intervals. Chemotherapy benefit was calculated as the difference in 5-year DRFI rates between the ET only and ET+CT groups. A multivariate Cox proportional hazards model was constructed to evaluate the association between chemotherapy treatment and DRFI, adjusting for MPI, age, menopausal status, tumor stage, lymph node status, and tumor grade. To assess whether the effect of chemotherapy varied by these covariates, the model included interaction terms between chemotherapy and each variable. Significance of interaction was tested using likelihood ratio tests comparing models with and without the interaction term. Statistical significance was defined as a 2-sided *P*-value of *P < *.05 for all tests. All statistical analyses were conducted using R (version 4.4.1) and SAS (version 9.4; SAS Institute, Inc., Cary, NC).

## Results

### Patient clinical characteristics

A total of 1002 propensity-score matched patients with HR+HER2- BC (*n* = 501 per treatment group) who were enrolled in the FLEX trial were included in the analysis (Table 1). Age, menopausal status, tumor stage, and nodal status were comparable between treatment groups. Overall, the cohort had a mean age of 59 years, with the majority being postmenopausal (70.1%) and White (79.2%). Most patients had either T1 (37.9%) or T2 (43.7%) tumors, were node-negative (70.0%), and had either G1 (25.1%) or G2 (51.2%) disease. Among the population, 12.2% had MammaPrint UltraLow, 35.8% had Low, 41.1% had H1, and 10.9% had H2 cancers. Patients treated with ET only were more likely to be White (85.4%), were less likely to be diagnosed with G3 tumors (6.4%), and were more likely to have MammaPrint Low Risk tumors (61.1%). In comparison, patients treated with ET+CT were less likely to be White (73.1%), were more likely to have G3 cancers (29.3%), and were less likely to have a MammaPrint Low Risk tumor (10.6%). The median follow-up for the ET only group was 4.97 years (Q1-Q3, 4.45-5.12), and it was 4.95 years (Q1-Q3, 4.42-5.14) for the ET+CT group.

**Table 1.** Matched clinical characteristics of FLEX patients with HR+HER2- tumors. Data represented as No. (%), unless otherwise specified. Patients were matched by menopausal status tumor stage, and nodal status. Differences in groups were assessed by using Pearson’s χ^2^ tests or Fisher exact tests (for categorical variables) or Student’s *t*-test (for numerical variables). Statistical significance was defined as *P < *.05. Patient self-identified race is listed.

Characteristic	ET only (*n* = 501)	ET+CT (*n* = 501)	All (*n* = 1002)	*P*
Age, years				
Mean (SD)	59 (± 12)	58 (± 11)	59 (± 12)	.12
Menopausal status				
Pre/Peri	141 (28.1%)	119 (23.8%)	260 (25.9%)	.319
Post	342 (68.3%)	360 (71.9%)	702 (70.1%)	
Unknown	18 (3.6%)	22 (4.3%)	40 (4.0%)	
Race				
American Indian or Alaska Native	0 (0%)	1 (0.2%)	1 (0.1%)	<.001
Asian, Asian American, or Pacific Islander	8 (1.6%)	16 (3.2%)	24 (2.4%)	
Black	24 (4.8%)	61 (12.2%)	85 (8.5%)	
Latin American	14 (2.8%)	29 (5.7%)	43 (4.3%)	
White	428 (85.4%)	366 (73.1%)	794 (79.2%)	
Unknown	27 (5.4%)	28 (5.6%)	55 (5.5%)	
Tumor stage				
T1	197 (39.3%)	183 (36.5%)	380 (37.9%)	.853
T2	208 (41.5%)	230 (45.9%)	438 (43.7%)	
T3	54 (10.8%)	47 (9.4%)	101 (10.1%)	
T4	6 (1.2%)	9 (1.8%)	15 (1.5%)	
Unknown	36 (7.2%)	32 (6.4%)	68 (6.8%)	
Lymph node status				
LN-	361 (72.0%)	341 (68.0%)	702 (70.0%)	.225
LN+	102 (20.4%)	125 (25.0%)	227 (22.7%)	
Unknown	38 (7.6%)	35 (7.0%)	73 (7.3%)	
Grade				
G1	185 (36.9%)	66 (13.2%)	251 (25.1%)	<.001
G2	259 (51.7%)	254 (50.7%)	513 (51.2%)	
G3	32 (6.4%)	147 (29.3%)	179 (17.8%)	
Unknown	25 (5.0%)	34 (6.8%)	59 (5.9%)	

Abbreviations: HR+ = hormone receptor-positive; HER2- = human epidermal growth factor receptor 2-negative; CT = chemotherapy; ET = endocrine therapy; *n* = number of participants; SD = standard deviation.

### Correlation between clinical features and MammaPrint index

**Figure 1.**
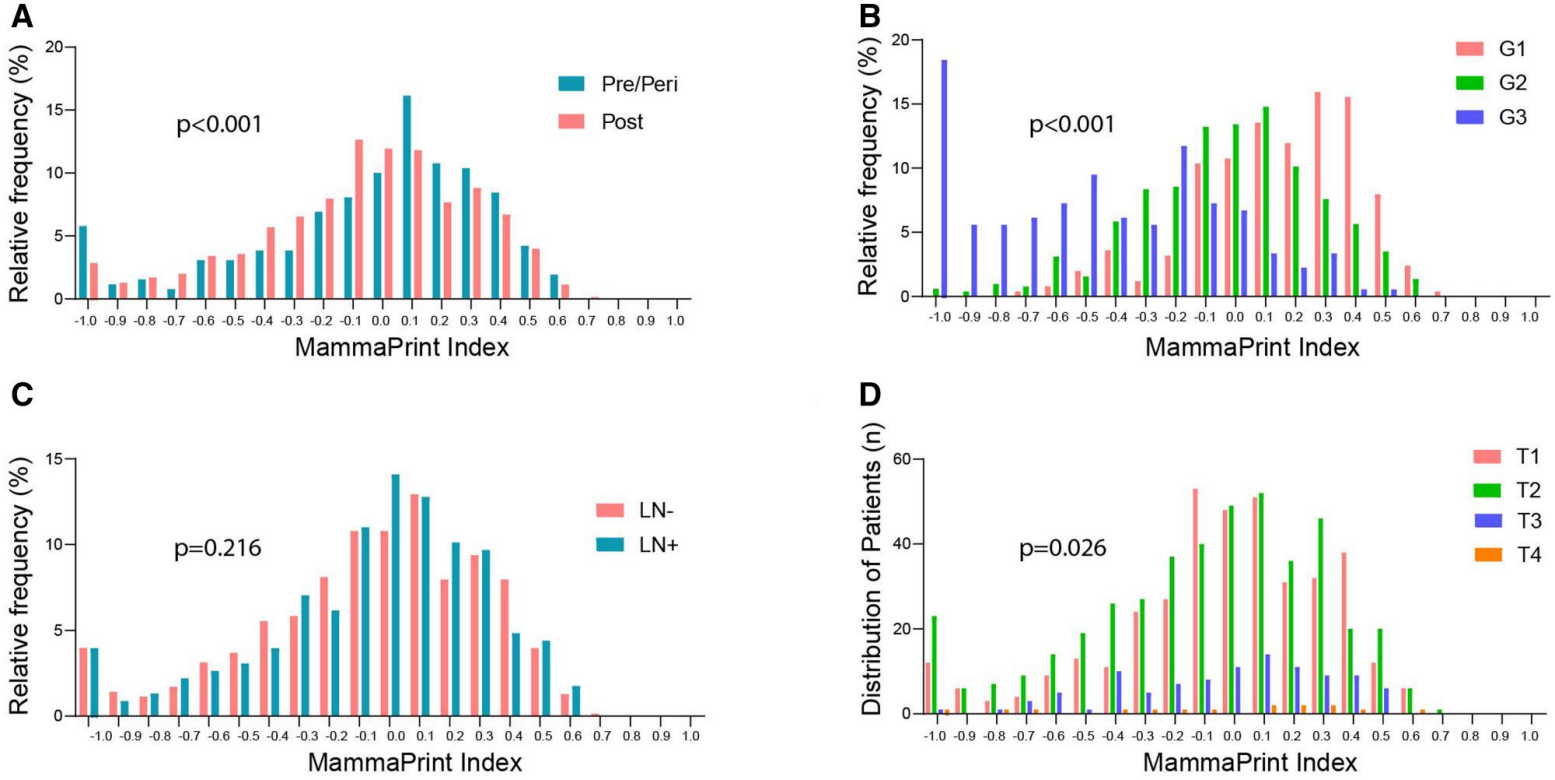
χ^2^ comparison of distribution of patients’ clinical characteristics across MammaPrint Index. Frequency of **A)** pre/peri- and postmenopausal status, **B)** tumor grade, **C)** lymph node status, and **D)** tumor stage across MammaPrint Index. Abbreviation: LN = lymph node.

A sensitivity analysis was conducted to examine the distribution of menopausal status, tumor grade, and nodal status across MammaPrint Index values (Figure 1). The distribution of menopausal status (*P < *.001), grade (*P < *.001), and tumor stage (*P = *.026) differed significantly across the MammaPrint Index, whereas nodal status did not (*P = *.216). Notably, a higher proportion of pre/perimenopausal patients (27.0%) had MammaPrint Index values in the Low Risk range of 0.1 to 0.2, compared with postmenopausal patients (19.5%), of which the greatest difference was observed at index 0.1 (Figure 1, A). The BCs classified as MammaPrint High Risk were more likely to be G3 (Figure 1, B), with a Cramer’s V test correlation coefficient of 0.88, indicating a strong association between the G3 and MammaPrint High Risk designations. However, 27.1% of the G1 cancers were either H1 or H2 Risk, and 10.6% of the G3 cancers were UltraLow or Low Risk. G2 cancers were evenly distributed between the High Risk (51.3%) and Low Risk (48.7%) subgroups. Similar proportions of patients with MammaPrint Low or UltraLow Risk cancers had nodal involvement (22.1%), compared with patients with H1 or H2 disease (23.3%; Figure 1, C). T-stages were relatively evenly distributed across the MammaPrint Index subgroups (Figure 1, D).

### MammaPrint and chemotherapy benefit

**Figure 2.**
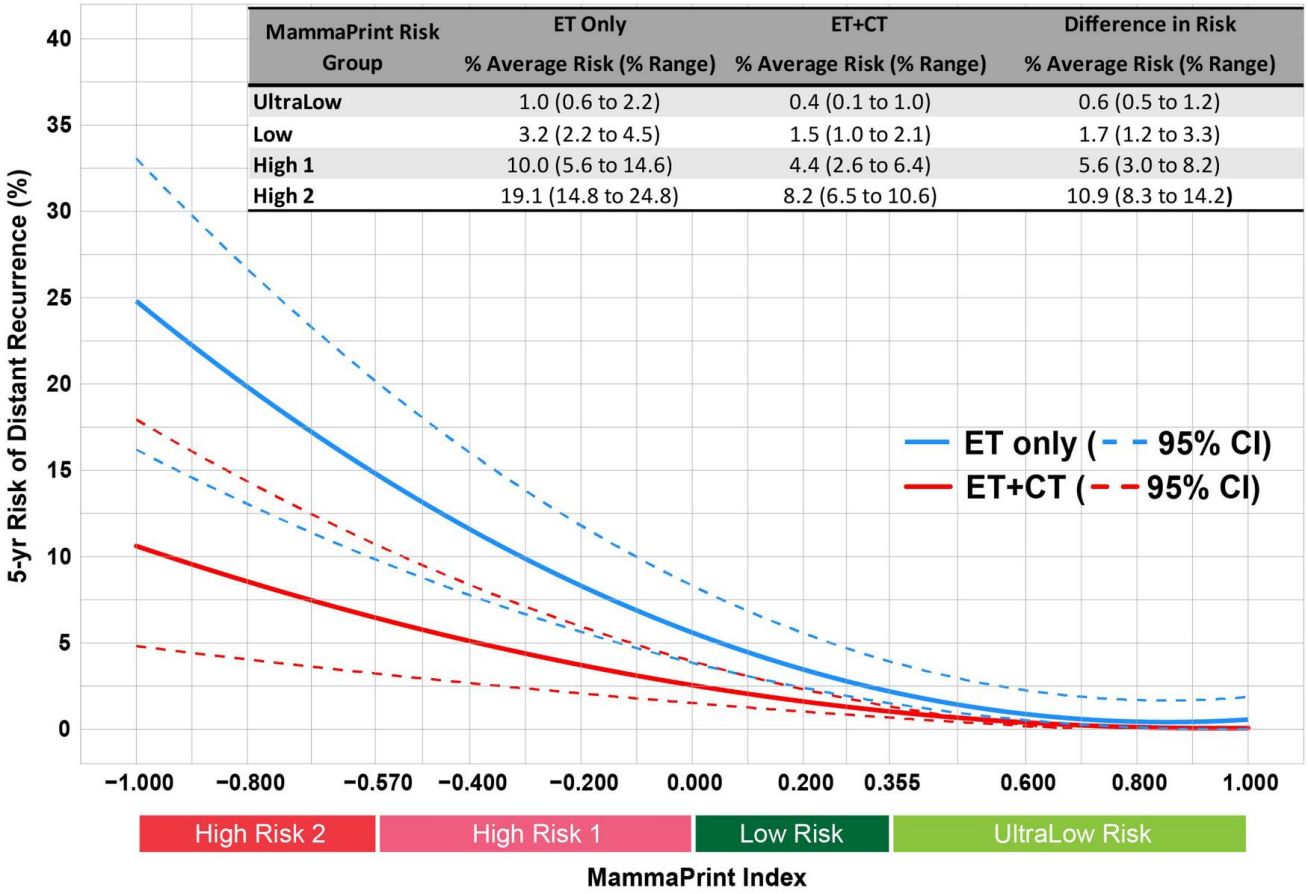
The MammaPrint Index and prediction of risk of distant recurrence as a continuous variable for HR+HER2- breast cancer patients. Likelihood of distant recurrence free interval (DRFI) event risk as a continuous function of the MammaPrint Index for ET only and ET+CT treated patients. The dashed curves indicate 95% CI for the respective treatment groups. The MammaPrint Index and Risk categories are denoted on the x-axis. Percent average and percent range of risk for each treatment and MammaPrint Risk groups are defined within the shaded box. Abbreviations: ET = endocrine therapy; CT = chemotherapy; CI = confidence interval.

Figure 2 illustrates the relationship between the MammaPrint Index and 5-year DRFI in the ET only and ET+CT groups. Regression model analysis demonstrated that the MammaPrint Index was highly predictive of DRFI outcome in both the ET only (R^2^ = 0.99, *P < *.001) and ET+CT groups (R^2^ = 0.90, *P < *.001). Among patients treated with ET only, the risk of a DRFI event increased significantly with higher MammaPrint Index Risk (Figure 2). For patients treated with ET only with UltraLow Risk cancers, 5-year risk of a DRFI event ranged from 0.6% to 2.2%, with an average of 1.0%. Patients with Low Risk cancers had a DRFI risk between 2.2% and 4.5% (average 3.2%), and those with H1 tumors had a DRFI risk ranging from 5.6% to 14.6% (average 10.0%). The highest DRFI risk was observed in patients treated with ET only with H2 tumors, with estimates ranging from 14.8% to 24.8% (average 19.1%). In the ET+CT group, patients with UltraLow Risk tumors had a 5-year DRFI risk between 0.1% and 1.0% (average 0.4%), whereas those with Low Risk cancers had a risk between 1.0% and 2.1% (average 1.5%). Among patients with H1 cancer, DRFI risk ranged from 2.6% to 6.4% (average 4.4%), whereas those with a H2 cancer had a DRFI risk between 6.5% and 10.6% (average 8.2%).

Significant reductions in the risk of a DRFI event in the ET+CT group compared with the ET only group were detected in H1 cancers, with an absolute benefit ranging from 3.0% to 8.2% (average: 5.6%) and increased with higher MammaPrint Risk. The greatest chemotherapy benefit was observed in patients with H2 tumors (8.3% to 14.2% absolute reduction in DRFI event rate; average: 10.9%). In contrast, chemotherapy provided minimal DRFI benefit in patients with Low Risk (average: 1.7%) or UltraLow Risk cancers (average: <1.0%).

**Figure 3.**
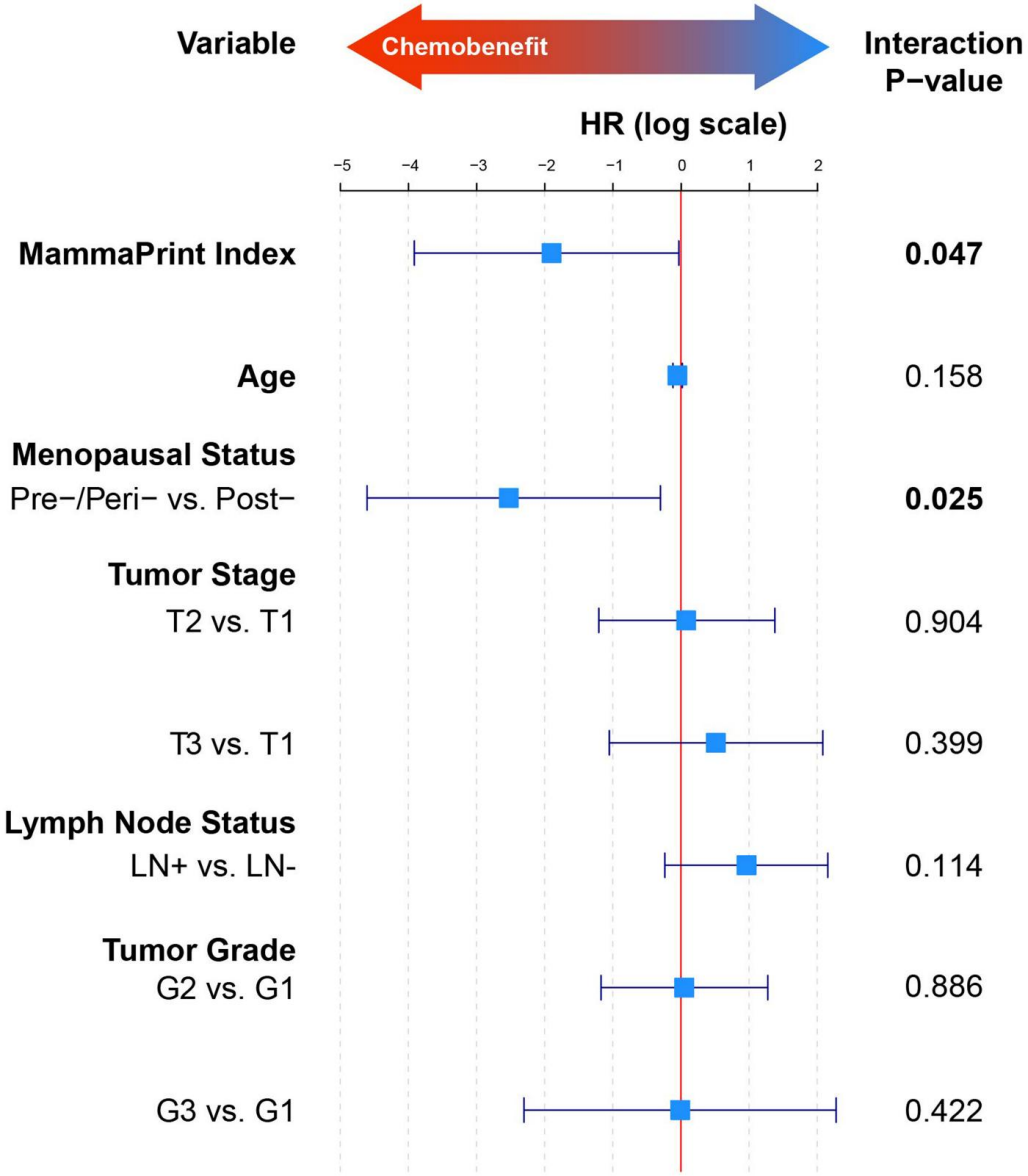
Likelihood ratio tests of the interaction of chemotherapy treatment with MammaPrint Index (MPI) and clinical variables. This figure shows the same data presented in Table 2, but on a natural logarithmic scale. Data represented as HR (95% CI, *P*-value). *P < *.05 indicates significant risk factor. A significant interaction term suggests that the effect of chemotherapy on DRFI varies according to the status of the indicated variable. In this table, the association between chemotherapy use and DRFI is influenced by MPI. The HR of 0.15 for MPI indicates that for each 1-unit change toward higher MPI Risk, patients derive significantly greater benefit from chemotherapy, with an 85% reduction in the risk of a DRFI event. Abbreviations: HR = hazard ratio; CI = confidence interval.

A multivariate Cox proportional hazards analysis was performed for 5-year DRFI to assess the interaction between treatment groups and genomic and clinical factors, including the MammaPrint Index as a continuous variable (Table 2 and Figure 3). There was a significant interaction between chemotherapy treatment and the MammaPrint Index (HR = 0.15; 95% CI = 0.02 to 0.97, *P = *.047), indicating that increasing MammaPrint Index Risk strongly predicts relative benefit of chemotherapy. Additionally, pre/perimenopausal status was significantly associated with chemotherapy benefit (HR = 0.08; 95% CI = 0.01 to 0.74, *P = *.025). However, age (HR = 0.95, 95% CI = 0.89 to 1.02, *P = *.158), tumor stage (T2: HR = 1.08, 95% CI = 0.30 to 3.97, *P = *.904; T3: HR = 1.67, 95% CI = 0.35 to 8.03, *P = *.399), positive nodal status (HR = 2.62, 95% CI = 0.79 to 8.63, *P = *.114), and higher-grade tumors (G2: HR = 1.05, 95% CI = 0.31 to 3.58, *P = *.886; G3: HR = 0.99, 95% CI = 0.10 to 9.76, *P = *.422) did not show significant interactions with treatment group in predicting chemotherapy benefit.

**Table 2.** Likelihood ratio tests of the interaction of chemotherapy treatment with MammaPrint Index (MPI) and clinical variables. Data represented as HR (95% CI, *P*-value). *P < *.05 indicates significant risk factor. A significant interaction term suggests that the effect of chemotherapy on DRFI varies according to the status of the indicated variable. In this table, the association between chemotherapy use and DRFI is influenced by MPI. The HR of 0.15 for MPI indicates that for each 1-unit change toward higher MPI Risk, patients derive significantly greater benefit from chemotherapy, with an 85% reduction in the risk of a DRFI event.

Variable	Adjusted HR multivariate	Interaction *P*
MammaPrint Index	0.15 (0.02 to 0.97)	.047
Age	0.95 (0.89 to 1.02)	.158
Menopausal status		
Post	Reference	
Pre/Peri	0.08 (0.01 to 0.74)	.025
Tumor stage		
T1	Reference	
T2	1.08 (0.30 to 3.97)	.904
T3	1.67 (0.35 to 8.03)	.399
Lymph node status		
LN-	Reference	
LN+	2.62 (0.79 to 8.63)	.114
Grade		
G1	Reference	
G2	1.05 (0.31 to 3.58)	.886
G3	0.99 (0.10 to 9.76)	.422

Abbreviations: HR = hazard ratio; CI = confidence interval.

## Discussion

Multi-gene expression assays play a key role in personalizing adjuvant chemotherapy treatment decisions for patients with HR+HER2- early-stage BC. MINDACT has proven that MammaPrint can identify clinically high risk, genomically low risk patients who have excellent outcomes without chemotherapy, but it was not specifically designed to evaluate MammaPrint as a predictor of chemotherapy benefit in MammaPrint High Risk patients. This study is the first prospective, real-world data analysis evaluating MammaPrint Index as a predictor of chemotherapy benefit in a propensity-score matched cohort of patients with HR+HER2- early-stage BC across the spectrum of clinical and genomic high and low risk.

Chemotherapy significantly reduced the risk of a DRFI event as MammaPrint Risk increased to H1 and H2, with an absolute chemotherapy benefit of up to 14.2% in patients with BC with an index of -1.0 in the H2 range. Notably, a significant interaction test (HR = 0.15; 95% CI = 0.02 to 0.97, *P = *.047) further supports the MammaPrint Index as a predictive marker of chemotherapy benefit in H1 and H2 HR+HER2- cancers. These findings align with retrospective, observational analyses pooled from 7 studies, which reported 541 patients with MammaPrint High Risk, early BC had a 5-year distant disease-free survival (DDFS) of 76% with ET only (*n* = 315), compared with 88% with ET+CT (*n* = 226), demonstrating a 12% absolute chemotherapy benefit (*P < *.01).^16^ Additionally, higher MammaPrint Index risk has been significantly associated with an increased probability of pathological Complete Response (pCR) with neoadjuvant chemotherapy.^17^ Analysis of the I-SPY2, NBRST, and FLEX trials further confirmed that MammaPrint H2 is associated with chemosensitivity, with pCR rates ranging from 22% to 29% in patients with HR+HER2- early-stage BC.^18-21^

Multivariate analysis also revealed that MammaPrint’s association with chemotherapy benefit was independent of other tumor characteristics in the FLEX trial. Most patients had T1 or T2, node-negative BC, with these features evenly represented across the MammaPrint Index. Importantly, tumors with lower risk clinical features (T1 or T2, node-negative) were well represented in the MammaPrint High Risk group and had demonstrated improvement in 5-year DRFI with chemotherapy. Although MammaPrint and tumor grade showed high collinearity, there was notable discordance between these factors. Specifically, 27% of G1 tumors were classified as MammaPrint High Risk, whereas more than 10% of G3 tumors were classified as UltraLow or Low Risk, suggesting that the 70-gene MammaPrint assay assesses tumor biology differently than morphological grading. Differences in subjective interpretation of clinical pathological features, which has been shown to be discordant among pathologists with rates of up to 42% across scoring sessions, may also explain the lack of agreement between grade and MammaPrint Risk in some patients.^22^ Importantly, although patients with G3 tumors were more likely to receive chemotherapy, tumor grade was not found to significantly predict chemotherapy benefit in a multivariate model.

Despite similar rates of nodal involvement compared with those with high risk tumors, patients with low risk disease did not derive significant chemotherapy benefit. These findings are consistent with the results from the MINDACT trial, where 21.2% (912/4294) of patients with low risk and 20.5% (492/2398) with high risk exhibit node positive status. The MINDACT trial confirmed that MammaPrint can accurately identify patients with clinically high risk, genomically low risk tumors who can safely forego chemotherapy and have excellent long-term outcomes.^4^^,^^5^

Interestingly, this study observed a small chemotherapy benefit (1.9%–2.4%) for patients within the low risk indices ranging from 0.001 to 0.200. This finding may be attributed to the observed higher proportion of pre/perimenopausal vs postmenopausal women in this low risk range, who have been shown to benefit from chemotherapy in previous studies in the absence of treatment with a GnRH agonist. Data reported from the TAILORx, MINDACT, and RxPONDER trials showed that premenopausal or younger (≤50 years) women, with HR+HER2-, genomic low risk BC benefited from chemotherapy with improved long-term outcomes, whereas low risk postmenopausal women did not. Indeed, in this report, multivariate analysis identified premenopausal status, but not age, as a factor associated with chemotherapy benefit. However, it has been suggested that this chemotherapy benefit in premenopausal women may be due to ovarian function suppression (OFS) from chemotherapy, rather than a direct antitumor effect.^23-25^ Emerging data support this OFS hypothesis. A post hoc analysis of the RxPONDER trial found that premenopausal patients with HR+HER2- BC and higher anti-Müllerian hormone (AMH) levels (≥10 pg/mL), indicating normal ovarian reserve, experienced benefit with the addition of chemotherapy to ET (HR = 0.48; 95% CI = 0.33 to 0.69) compared with patients with lower AMH levels (<10 pg/mL (HR = 1.21; 95% CI = 0.60 to 2.43), indicating that the amenorrhea as a result from chemotherapy may provide the observed clinical benefit.^26^ Further supporting this hypothesis, the TAILORx study reported no significant chemotherapy benefit among postmenopausal patients aged 46-50 years^27^—an observation that may explain why age was not significantly associated with chemotherapy benefit in the multivariate analysis of this study. Furthermore, whole transcriptome analysis comparing HR+HER2- early-stage BC from patients ≤50 vs >50 years from the FLEX trial found no substantial differences in gene expression in MammaPrint Low Risk, genomically luminal tumors. Together, these results indicate that age-related differences in chemotherapy benefit may be driven by the OFS effect of chemotherapy in younger women.^28^

A key strength of the present study includes applying propensity-score matching within a diverse real-world patient population to demonstrate the value of the MammaPrint Index in predicting chemotherapy benefit. The well-matched cohort of 1002 patients with HR+HER2- early-stage BC allowed for direct comparisons between ET only and ET+CT groups, minimizing the impact of confounders. Additionally, this is the first prospective, RWD analysis that demonstrates MammaPrint Index as a strong predictor of chemotherapy benefit. The use of RWD captures a more representative proportion of the patient population than controlled trials, improving the generalizability of the findings. Lastly, the prospective design of FLEX, unlike most RWD studies, increases the quality of the data that are collected and queried in real time.

Limitations of this study include the need for longer follow-up beyond 5 years and the observational, nonrandomized nature of the study, which may introduce biases in patient selection or physician treatment choices. Although most patients were enrolled after 2018, when the SOFT and TEXT trials (NCT00066690, NCT00066703) established the efficacy of OFS combined with ET in premenopausal HR+ BC, the FLEX registry captures limited data on the use of OFS and CDK4/6 inhibitors, both of which now represent standard treatment for early-stage BC. Future analysis is warranted to investigate outcomes associated with newer treatments. Due to evolving treatment standards, conducting RCTs to evaluate MammaPrint’s utility in predicting chemotherapy benefit in clinically low and high risk patients is not feasible, and archival tissue from historical RCTs assessing chemotherapy benefit is not available.^6^^,^^7^ Thus, observational RWD analyses that apply accepted statistical methods, including propensity-score matching, highlight the value in novel decentralized databases that can provide actionable RWE that can inform clinical practice. Importantly, the outcomes observed here align with the results from MINDACT^4^^,^^5^ and are consistent with previous retrospective analyses of randomized^29^ and nonrandomized^30^ early-stage BC trials, which have demonstrated chemotherapy benefit in high-risk patients using other genomic assays.

## Conclusions

This RWD analysis of the prospective FLEX registry demonstrates a significant chemotherapy benefit in patients with MammaPrint High Risk, HR+HER2- early BC within a propensity score-matched cohort. These data further confirm MammaPrint’s utility in identifying patients with low risk cancers who can safely forego chemotherapy. These findings add to the evidence from MINDACT and further support the utility of MammaPrint as both a predictive and prognostic tool in assessing the likelihood of chemotherapy benefit in HR+HER2- early-stage BC.^4^^,^^5^

## Supplementary Material

pkaf079_Supplementary_Data

## Data Availability

Data are available from the corresponding author upon reasonable request.
