# Supplementary material for: MammaPrint predicts chemotherapy benefit in HR+HER2- early breast cancer: FLEX Registry real-world data
Source: JNCI Cancer Spectr. 2025 Aug 11;9(5):pkaf079. doi: 10.1093/jncics/pkaf079 (PMC12413225; doi:10.1093/jncics/pkaf079)
Supplement: pkaf079_Supplementary_Data [file pkaf079_supplementary_data.zip › Supplementary Table S1_15Jul25.pdf]

Supplementary Material

Characteristic	ET only (n=859)	ET+CT (n=548)	All (n=1407)	P-Value
Age (Years)				
Mean (SD)	62 (± 11)	57 (± 12)	60 (± 11)	<0.001
Menopausal Status				
Pre-/Peri-	141 (16.4%)	154 (28.1%)	295 (21.0%)	<0.001
Post-	673 (78.3%)	368 (67.2%)	1041 (74.0%)	
Unknown	45 (5.2%)	26 (4.7%)	71 (5.0%)	
Race				
American Indian or Alaska Native	0 (0%)	1 (0.2%)	1 (0.1%)	<0.001
Asian, American, or Pacific Islander	12 (1.4%)	17 (3.1%)	29 (2.1%)	
Black	43 (5.0%)	66 (12.0%)	109 (7.7%)	
Latin American	32 (3.7%)	32 (5.8%)	64 (4.5%)	
White	724 (84.3%)	402 (73.4%)	1126 (80.0%)	
Unknown	48 (5.6%)	30 (5.5%)	78 (5.5%)	
Tumor Stage				
T1	387 (45.1%)	203 (37.0%)	590 (41.9%)	0.0085
T2	325 (37.8%)	231 (42.2%)	556 (39.5%)	
T3	80 (9.3%)	69 (12.6%)	149 (10.6%)	
T4	6 (0.7%)	13 (2.4%)	19 (1.4%)	
Unknown	61 (7.1%)	32 (5.8%)	93 (6.6%)	
Lymph Node Status				
LN-	102 (11.9%)	160 (29.2%)	262 (18.6%)	<0.001
LN+	680 (79.2%)	352 (64.2%)	1032 (73.3%)	
Unknown	77 (9.0%)	36 (6.6%)	113 (8.0%)	
Grade				
G1	333 (40.6%)	75 (14.5%)	408 (30.5%)	<0.001
G2	437 (53.3%)	278 (53.8%)	715 (53.5%)	
G3	46 (5.6%)	159 (30.8%)	205 (15.3%)	
Unknown	4 (0.5%)	5 (1.0%)	9 (0.7%)	

Table S1. Clinical characteristics of FLEX patients with HR+HER2- tumors (unmatched). Data represented as n (%), unless otherwise specified. Differences in groups were assessed by using Pearson's Chi-squared test, Fisher's exact test (for categorical variables), or Student's t-test (for numerical variables). Statistical significance was defined as p<0.05. Abbreviations: HR+, hormone receptor-positive; HER2-, CT, chemotherapy; ET, endocrine therapy; human epidermal growth factor receptor 2-negative; n, number of participants; SD, standard deviation.
